# An automated system based on 2 d empirical mode decomposition and k-means clustering for classification of *Plasmodium species* in thin blood smear images

**DOI:** 10.1186/1471-2334-14-S3-P13

**Published:** 2014-05-27

**Authors:** K Manickavasagam, S Sutha, K Kamalanand

**Affiliations:** 1 Department of Instrumentation Engineering, MIT Campus, Anna University, Chennai, India

## Background

In order to control malarial infection, specific anti-malarial drug for the corresponding *Plasmodium species* must be administered. The objective of this work is to develop a system for classification of *Plasmodium* species in thin blood smear images.

## Methods

In this work, thin blood smear sub images (n=87) of different *Plasmodium* species were acquired from the Parasite Image Library of the Centers for Disease Control and Prevention Database [http://www.dpd.cdc.gov/dpdx/HTML/ImageLibrary/Malaria_il.htm]. The images were subjected to 2 d Empirical Mode Decomposition and four features namely the mean value of first Intrinsic Mode Function (IMF-1), IMF-2, IMF-3 and residue, were extracted. The significance of the extracted features was analyzed using ANOVA test. Further, the k-means clustering algorithm was used to classify the different *Plasmodium* species using the significant features.

## Results

It was found that the features namely the mean of IMF-1 and residue are statistically significant (*p*<0.001) and the developed classification system was able to classify the *Plasmodium vivax* images with a high accuracy of 100%. Further, the *Plasmodium malariae* images were classified with an accuracy of 83.33%. However, the developed classifier showed lower accuracy in classification of *Plasmodium falciparum* and *Plasmodium ovale* images.

## Conclusion

Results demonstrate that the developed system is highly efficient in classification of *P. vivax* and *P. malariae*. This study appears to be of high clinical relevance since the automated classification of malaria parasite is useful for mass screening and drug selection for treatment of the infection.

